# The siren song of vocal fundamental frequency for romantic relationships

**DOI:** 10.3389/fpsyg.2013.00439

**Published:** 2013-07-15

**Authors:** Sarah Weusthoff, Brian R. Baucom, Kurt Hahlweg

**Affiliations:** ^1^Clinical Psychology, Psychotherapy, and Assessment, Department of Psychology, Technische Universität BraunschweigBraunschweig, Germany; ^2^Department of Psychology, University of Utah, Salt Lake CityUT, USA

**Keywords:** fundamental frequency, attraction, emotional arousal, romantic relationships, couples, communication, conflict

## Abstract

A multitude of factors contribute to why and how romantic relationships are formed as well as whether they ultimately succeed or fail. Drawing on evolutionary models of attraction and speech production as well as integrative models of relationship functioning, this review argues that paralinguistic cues (more specifically the fundamental frequency of the voice) that are initially a strong source of attraction also increase couples’ risk for relationship failure. Conceptual similarities and differences between the multiple operationalizations and interpretations of vocal fundamental frequency are discussed and guidelines are presented for understanding both convergent and non-convergent findings. Implications for clinical practice and future research are discussed.

## INTRODUCTION

Across cultures, adults rate marriage and long-term monogamous relationships as the most important ones in their lives (Buss, 2005). Despite this, divorce rates in industrialized countries rank between 40 to 55% (Hahlweg et al., 2010). A tremendous amount of effort has been accordingly devoted to identifying and understanding risk factors for relationship dissolution (Gottman and Notarius, 2002) with a particular emphasis on identifying risk factors that are present from the outset of a relationship (Caughlin and Huston, 2002). The polarization model of romantic relationships (Jacobson and Christensen, 1998; Baucom and Atkins, 2013) suggests that one of the most important kinds of risk factors originates in variables that are initially associated with attraction and desire that later become sources of distress and interpersonal friction. A growing body of empirical evidence suggests that the fundamental frequency of romantic partner’s voices may represent precisely this kind of risk. Vocal qualities that are found to be attractive during the early stages of relationship formation are also associated with increased levels of dysfunction, increased risk for divorce, and decreased likelihood of benefitting from couple therapy in later stages of a relationship.

Evolutionary models suggest that perceptions of health status and likelihood of reproductive success are one of the primary determinants of attractiveness and mate selection. Physical aspects of attractiveness like facial symmetry, body height, and the voice are indicative of levels of sexual hormones, and thus of the likelihood of producing healthy offspring. Generally, indicators of higher levels of masculinity in males (based on higher levels of testosterone) and higher levels of femininity in females (based on higher levels of estrogen) are perceived as more attractive and are linked to better reproductive success. This leads to a dimorphism in attractiveness ratings: males tend to prefer female partners with higher f_0_ voices and smaller body sizes while female tend to prefer males with lower f_0_ voices and larger body sizes (Puts et al., 2012). Though these sex-related differences are important determinants of attraction, the polarization model of relationship distress suggests that they may become associated with dysfunction and increased risk for divorce as relationships mature. A change from attraction to distress is often accompanied by a change in attribution for the difference itself. Differences that are seen as enriching and complimentary are often experienced as desirable (Aron and Aron, 1997) while differences that are seen as short-comings and faults in the other commonly contribute to a cycle of distress. When differences are seen as faults in the other, spouses typically alternate between criticizing and blaming one another for the problems in their relationship and defending themselves from their partner’s attacks (i.e., they engage in a strong demand/withdraw cycle of conflict). As this process unfolds, the relationship becomes polarized, and the three core aspects of interaction, communication, perception, and physiology, become imbalanced (Burman and Margolin, 1992; Gottman, 1993), conflict ensues, and even routine interaction with the spouse becomes highly aversive. The aversive nature of this cycle typically results in increasing polarization over time and makes it very difficult for spouses to resolve even minor problems. Being stuck in the polarization cycle is highly distressing for spouses, and high levels of aversive arousal during interaction are one of the most robust predictors of risk for relationship distress and dissolution (Gottman and Levenson, 1992). Aversive arousal has most often been captured via well-established physiological indices like heart rate (HR), blood pressure (BP), or skin conductance (Larsen et al., 2008) as well as via endocrine measures like cortisol, or epinephrine (Robles and Kiecolt-Glaser, 2003; Ditzen et al., 2011). In addition to well-replicated findings based on physiological measures, a growing body of evidence shows that higher levels of vocally encoded emotional arousal, which are reflected in higher levels of f_0_, are similarly related to increased risk for a wide range of negative relationship outcomes.

### FUNDAMENTAL FREQUENCY (f_0_)

As a mathematical quantity, fundamental frequency (f_0_) refers to the lowest frequency harmonic of the speech sound wave. This frequency is biologically determined by the pattern of vibration created by the vocal folds during phonation, the phase of speech production where the outward flow of air from the lungs is regulated by the larynx. Higher rates of opening and closing of the vocal folds across the glottis are associated with higher f_0_ values (measured in cycles per second or Hertz [Hz]). Perceptually, f_0_ is highly correlated with pitch where higher f_0_ values correspond to higher pitch (Juslin and Scherer, 2005). F_0_ can be easily calculated using Praat, a freely available, Windows-based software package (Boersma and Weenink, 2013; www.praat.org), to analyze existing audio recordings of speech in adequate quality. F_0_ is assessed continuously during human speech and can change rapidly. Different parameters like mean f_0_, f_0_ range, minimum f_0_, or maximum f_0_ can be calculated and used in empirical research. All of those can be calculated either at a very small scale (such as for each talk turn) or averaged across over the entirety of a conversation. As is true of much behavioral research, there is greater agreement about how to calculate f_0_ than there is about how to understand what f_0_ represents. F_0_ has been variously interpreted as an index of vocally encoded emotional arousal (see Juslin and Scherer, 2005 for a review; Weusthoff et al., 2013) and dominance (Puts et al., 2006; Borkowska and Pawlowski, 2011), and additional work demonstrates that f_0_ correlates with other factors such as age and pubertal (Hollien and Shipp, 1972; Brown et al., 1991; Hollien et al., 1994, 1997), phonemic and syntactic structure of speech (Whalen and Levitt, 1995). Recent work examining f_0_ during social interaction provides a framework for integrating the various interpretations of f_0_. In Weusthoff et al.’s 2013 examination of simultaneous associations between f_0_, biological sex, physiological indices of arousal, and social behavior, biological sex was the largest predictor of individual differences in f_0_ while physiological arousal and social behaviors were the best predictors of variance in f_0_ attributable to a specific social interaction. These results suggest that f_0_ can be understood as conveying information about both traits (such as biological sex) and states (momentary physiological arousal and social behavior) and highlights the need for careful analysis of f_0_ to allow for specific interpretations.

### METHOD OF REVIEW

In contrast to the nascent body of research examining f_0_ during marital interactions,f_0_ has been intensively studied using different research paradigms across multiple disciplines (e.g., single conversations recorded during naturally occurring stressful events like the New York City blackout in 1977, Streeter et al., 1982, or emotion portrayals by professional actors, Banse and Scherer, 1996; see Scherer, 2003 for a review). For example, a key-word based search on SCOPUS (using the search terms “social interaction” AND “verbal” OR “non-verbal” in “Keywords, Abstract, or Title”) yielded more than 2000 results. Here, we focus on research with a specific focus on relationship formation and maintenance. The body of work on f_0_ and attraction is robust and well-developed. Thus, we seek to provide a representative sampling of the main findings in this area. In contrast, there are many fewer studies of naturalistic social interaction in general much less about marital interaction specifically. As others have noted, studies using naturalistic vocal expressions of affect with sufficient intensity of the emotions displayed are needed in vocal expression research in order to obtain speech that offers sufficient levels of “ecological validity” (Juslin and Scherer, 2005). Communication in close relationships seems to fulfill these criterions but research has only recently begun to take f_0_ into account here.

Research with human participants can be split up in two further groups: whether interlocutors had some form of relationship with each other before interacting, or not. In the case of pre-existing relationship, four main areas of relationships have been covered: the ones between parents and infants (see Irwin, 2003 for a review), between psychotherapists and clients (see Greenberg and Pascual-Leone, 2006 for a review), between physicians and patients (see Hassan et al., 2007; Hulsman et al., 2011 for a review), and between spouses in intimate relationship. Independent of targets, f_0_ has been found to be associated with arousal. As it it beyond the scope of this review to cover this all, the interested reader is referred to the according reviews. In couples interactions, however, the role of vocally encoded emotional arousal has not been investigated closely. This review aims at looking into f_0_ and its associations with couple functioning more closely. The authors identified a total number of *N* = 5 studies for this task. All analyses were based on dyadic communication settings (conflict discussions) between spouses from long-term or married heterosexual couples. Instructions for the tasks were standardized, and recordings of the interactions were videotaped during assessment sessions conducted in research laboratories. Participants were either English (Baucom et al., 2009, 2011), or German (Baucom et al., 2012b; Kliem et al., 2012; Weusthoff et al., 2013) native speakers (for a detailed description of the studies included in this review, please see **Table 1**).

**Table 1:** Methodological details of interaction studies reviewed.

Study	participants and communication setting	design and independent measures (covariates)	dependent measures	relevant findings
Baucom et al. (2009)	130 heterosexual, US-American couples in a couple therapy study; conflict discussion with spouse in laboratory (10 min)	longitudinal; sociodemographics, intrapersonal predictors, communication behavior, interpersonal predictors; all assessed at pre-treatment	response to treatment 2 years after treatment termination (deteriorated, unchanged, improved, recovered)	Across therapies, lower levels of wives’ f_0_ range linked to a higher likelihood for being in recovered category at the two-year follow-up.
Baucom et al. (2011)	heterosexual, US-American couples (*n* = 130 in a couple therapy study, *n* = 38 distressed community couples); conflict discussion with spouse in laboratory (10 min)	cross-sectional; mean f_0_, relationship satisfaction, influence tactics	demand/withdraw behavior and role	Higher levels of demand/withdraw-behavior were independently linked to higher levels of mean f_0_ across gender. Demanders experienced higher levels of mean f_0_, compared to withdrawers.
Baucom et al. (2012b)	49 heterosexual, German couples participating in couple-relationship education (CRE); conflict discussion with spouse in laboratory (15 min)	longitudinal; f_0_ range (biological sex, relationship satisfaction)	number of skills taught in the program remembered 11 years after participation in CRE by spouses	Higher levels of pre-training f_0_ range were associated with fewer skills remembered 11 years after the training. Women remembered more skills than men.
Weusthoff et al. (2013)	67 heterosexual, German couples participating in CRE; conflict discussion with spouse in laboratory (15 min)	cross-sectional; psychophysiology, communication behavior, biological sex	f_0_ range	Higher levels of f_0_ range significantly linked to higher levels of physiological arousal, more negative and less positive communication behavior, and being female
Kliem et al. (2012)	68 heterosexual, German couples participating in CRE; conflict discussion with spouse in laboratory (15 min)	longitudinal; f_0_ range, relationship satisfaction, psychophysiology, communication behavior, depressive symptoms	relationship status 11 years after participation in CRE	For females, higher levels of f_0_ range significantly linked to a higher risk for relationship dissolution. For males, higher levels of diastolic blood pressure linked to higher risk for relationship dissolution.

As also described in Weusthoff et al. (2013), f_0_ was obtained continuously from the speech of each person during the problem discussion of the assessment point(s). Conversations were segmented per speaker using audio editing programs like Adobe Premier Pro, resulting in data per speaker containing only conversation parts during which he or she talked separately without any other human, or background sound being present. Bandpass filtering was applied to all voice samples prior to further analyses (calculating f_0_ values) in order to restrict f_0_ values to the normal range of emotional adult speech. The typical range for male speakers is 75–150Hz, for female speakers 150–300Hz, though wider limits can be observed during highly aroused emotional states (Owren and Bachorowski, 2007). Common f_0_ mean values are around 225 Hz for women, and about 120 Hz for men. Across the lifespan, women’s mean f_0_ decreases while male speakers’ mean f_0_ scores also decrease first, but start to rise again. Changes in adults are most prominent for both sexes between age 50 and 60, with women additionally experiencing hormonal influences associated with the onset of the menopause (Hollien and Shipp, 1972; Brown et al., 1991; Hollien et al., 1997). Though it is possible for human speakers to generate sound outside the typical filtering limits of 75 and 300 Hz, f_0_ scores outside of this range are very likely resulting from background machine or electronic noise. Minimum, maximum, and mean f_0_ values were generated separately for each spouse by analyzing their respective segmented audio recordings using Praat, a free multiple platform program (Boersma and Weenink, 2013). F_0_ range was calculated separately for each partner at each assessment by subtracting the partner’s averaged minimum f_0_ from the partner’s averaged maximum f_0_ across the whole conversation. In Baucom et al.’s (2011) study, f_0_ mean was derived from raw f_0_ scores across the whole conversation. For a more detailed description of potential f_0_ range indices, please see **Table 1** in Baucom et al. (2012a).

### f_0_ IN INTIMATE RELATIONSHIP RESEARCH

Due to three reasons, it is important to further investigate the role of f_0_ in intimate relationships. One, relationship deterioration and/or separation are associated with tremendous costs for affected individuals and society as a whole, both concurrently and in the long run. Dysfunctional relationships lead to poorer mental and physical health in spouses (Kiecolt-Glaser and Newton, 2001), a higher likelihood for divorce and reduced levels of social support (Amato, 2010). Due to higher levels of unhealthy behaviors like smoking, even a decreased life expectancy (Larson and Halfon, 2013) in affected children has been observed. Second, couple-relationship education programs (CRE) aim at reducing the risk of relationship dissolution by teaching communication skills and ways of dealing with aspects of couple’s emotional lives, e.g., *EPL – Ein Partnerschaftliches Lernprogramm* (“A Learning Program for Couples”; Kaiser et al., 1998), especially during conflict discussions. These programs are known to be highly effective in doing so both in the short and in the long run (Hawkins et al., 2008; Hahlweg and Richter, 2010). However, the mechanisms of change in CRE and how emotional arousal affects the work in and outcome of CRE are still unknown. Communication is assumed to be the central mechanism of change, however, empirical studies using the paradigm of couples having videotaped conflict discussions that are later rated and / or analyzed with regard to different aspects of behavior have not consistently supported this supposition. One reason for this might be the rather simplistic ways of operationalizing a complex behavior like human communication as single elements like positive, or negative utterances (Christensen, 2010). Therefore, research should consider several aspects of communication simultaneously in a multi-channel approach (Gottman and Notarius, 2002). Third, though emotional arousal is known to play a crucial role in relationship dissolution (Gottman and Levenson, 1992), the specific pathways and interactions with other important variables like communication behavior, or relationship satisfaction are still unknown (Weusthoff et al., 2013). As future research in this field should focus on how to “modify emotion-driven, dysfunctional, and destructive interactional behavior,” and to “elicit avoided, emotion-based” behavior (Christensen, 2010, pp. 36-37), there is additional need for an objective tool for detecting and explaining emotional arousal in the context of couple interactions and intimate relationships.

During courtship, f_0_ is one of the evolutionary most important signals for non-visual gender discrimination (which is important for successful identification of potential mates; Junger et al., 2013), and for judging the attractiveness of a potential spouse (Borkowska and Pawlowski, 2011). Very masculine voices in males (perceived as low voice pitch, and thus low f_0_), and very feminine voices in females (perceived as high voice pitch, and thus high f_0_) are perceived as particularly attractive (DeBruine et al., 2006; Feinberg et al., 2006, 2012; Borkowska and Pawlowski, 2011; O’Connor et al., 2012). These perceptions are attenuated in both men and women during high-fertile phases of the female menstrual cycle (Puts, 2005; Pipitone and Gallup, 2008; Hodges-Simeon et al., 2010). High vocal masculinity in males are linked to high levels of testosterone, and associated with a higher likelihood for producing healthy offspring (O’Connor et al., 2012). However, in the long run, males with higher levels of vocal masculinity report a larger number of different sexual partners, and are less likely to engage in relationship maintaining and parental behaviors (O’Connor et al., 2011, 2012). It also seems plausible that f_0_ during relationship initiation stages displays high levels of arousal that is taken by partner as an indicator for higher engagement in a relationship, also hinting at a process similar to polarization.

During maintenance, less research on f_0_’s role has been conducted so far. F_0_ has most often been studied in couples’ conflict interactions where it seems to index levels of emotional arousal. F_0_ during couple conflict has been demonstrated to be significantly positively associated with multiple cardiac, and endocrine indices of arousal, and linked to more negative and less positive communication behavior. F_0_ is thought to simultaneously display autonomic physiological as well as socially learned reactions in one signal (Weusthoff et al., 2013). Furthermore, it has been shown that spouses influence each other in their levels of arousal. If one partner is highly aroused, it is becomes more difficult for the other one to maintain on a functional level of arousal, thus leading to polarization processes with regard to f_0_ (Baucom and Atkins, 2013).

As also noted elsewhere (Weusthoff et al., 2013), f_0_ offers a number of methodological and conceptual benefits with regard to being further used in research on romantic relationships. Its non-invasive nature and lack of need for additional equipment (only a good audio recording device is needed) makes the assessment of f_0_ an excellent candidate for data collection in situations like conflict interactions that are most often videotaped, or for post-hoc analyses (e.g., spousal discussions, or therapy sessions). Additionally, f_0_ can be computed and analyzed from the videotapes even after conducting a study in which it was not of primary interest. Furthermore, f_0_ is more closely associated with individual psychological than physical distress (Johannes et al., 2007), making it more sensitive to detect changes in psychological load and less prone to artifacts that influence physiological measures of arousal like HR or skin conductance (e.g., movements, Sloan and Kring, 2007). Being based on physical properties of speech, f_0_ can be analyzed objectively and independent of the researcher’s native language and culture (Weusthoff et al., 2013). Perhaps most importantly, f_0_ is directly involved in and available during the process of communication and can be perceived by a listener (Baucom et al., 2011). There is good evidence that partners in communication directly respond to each other’s f_0_ without being aware of this fact (Gregory and Webster, 1996). Similar to cardiovascular indices of arousal (e.g., HR), f_0_ seems to encode aspects of emotional arousal that are part of an autonomic process not entirely controllable by a speaker, similar to conditioned emotional responses (Kliem et al., 2012).

Merging these theories, expression of emotions (emotional expression) toward one’s spouse can be considered as a central part of well-functioning intimate relationships, the role of underlying emotional arousal, however, seems to be somewhat different. Endocrine and psychophysiological indices of heightened levels of emotional arousal have consistently been linked to unwanted relationship outcomes like dysfunctional communication behavior, higher risk for divorce and separation, or lower levels of relationship satisfaction (Gottman and Levenson, 1992; Kiecolt-Glaser and Newton, 2001; Gottman and Notarius, 2002). As emotions and their underlying processes like arousal are essential parts of social interactions (Juslin and Laukka, 2003) and can influence them heavily (Scherer, 2005), it makes them important information not only for researchers in the social interaction area but also for people’s everyday lives.

## STUDIES REVIEWED

### SUMMARY OF FINDINGS

Across studies, languages, and gender, f_0_ has been treated as an index of emotional arousal. Like other indices f_0_ has been associated with a number of different negative aspects of couple functioning both concurrently and in the long run. Significant associations have been found in conflict interactions of distressed couples, either participating in couples therapy, or in CRE. More specifically, higher levels of a person’s physiological indicators of emotional arousal like HR, BP, or salivary cortisol were associated with higher levels of one’s own f_0_. Higher levels of a spouse’s f_0_ have also been linked to higher levels of negative communication behavior, both observed and self-reported, and to lower levels of observed non-verbal positive communication behavior. In highly distressed couples, the likelihood for long-term success of couple therapy (being in the recovered range 2 years after treatment termination) was higher when wives’ displayed lower levels of f_0_ in a conflict discussion prior to treatment. Elevated levels of f_0_ in conflict discussions prior to participation in CRE also lead to a higher likelihood for separation and divorce, and a smaller number of communication skills remembered 11 years after the CRE.

## DISCUSSION

This review aimed at examining research on vocally encoded emotional arousal, namely f_0_, in close personal relationship communication (interaction between spouses). Significant associations were found with psychophysiological and endocrine indices of emotional arousal, observed and self-reported communication behavior, and relationship functioning (stability and satisfaction). Furthermore, f_0_ has been found to be related to skills remembrance in CRE. Significant results were found for concurrent as well as longitudinal links between f_0_ and variables of interest, and for a time frame as long as 11 years. Significant gender differences emerged for f_0_ range’s association with some variables of interest but not for f_0_ mean’s.

### FINDINGS CONSIDERED FROM AN EVOLUTIONARY PERSPECTIVE

Across studies, f_0_ was analyzed as an index of emotional arousal thought to display information about the internal state of the speaker to an interaction partner via the human voice. Stable associations between f_0_ and different forms of communication behavior (positive, negative, observed, self-reported, verbal, and non-verbal) emerged across studies. Social signaling theories assume evolutionary reasons for this: In order to heighten chances of survival in ancient hunter-gatherer societies, emotions were adaptive developments helping to avoid danger and to cooperate with others (Buss, 2005). Vocally encoded emotional arousal enables individuals to communicate emotions non-verbally from one person to another, independent of words and language. Vocal communication of emotion is more likely to appear in goal-relevant behavior (Juslin and Laukka, 2003). F_0_ scores indexing vocally encoded emotional arousal should therefore be higher during goal-relevant behavior. What is considered as goal-relevant behavior depends on situational aspects: Couple and family conflict is conceptually thought of as a chronic stressor (Doss et al., 2004) where often a wide range of negative communication behavior (like DW) is displayed in order to achieve change in one’s spouse (Christensen et al., 2006) and high levels of emotional arousal are displayed via multiple channels (Kiecolt-Glaser and Newton, 2001). Positive associations between f_0_ and these variables as found in the reviewed studies investigating conflict could be indexing goal-achieving strategies by spouses.

With regard to the theoretical foundation of emotional arousal, coordinated responses in multiple physiological systems stem from the same source, namely the cognitive appraisal in a given situation and the following changes in autonomous and somatic nervous system activity (Scherer, 2009). These changes were assessed via multiple channel of arousal in Weusthoff et al.’s 2013 study, with the associations between f_0_ and psychophysiological indices of arousal being in expected directions. With the periaqueductal grey (PAG), a brain-stem based areal (and thus an evolutionary old one) in humans is involved in both vocal and cardiovascular responses to different kinds of stress. The PAG is thought to integrate emotional aspect of stress responses into the autonomic nervous system responses in different channels (Linnman et al., 2012). These findings suggest that f_0_ as an index of emotional arousal, and thus the intensity of the emotional reaction (Weusthoff et al., 2013), are influenced by both basic biological processes as well as by socially learned communication behaviors stemming from a similar evolutionary basis (Juslin and Laukka, 2003).

Different brain regions are found to be involved in processing male and female voices (Sokhi et al., 2005), and discriminatory performance for human voices was better for opposite-sex stimuli than for same sex-stimuli (faster identification; Junger et al., 2013). F_0_ seems to enable a stronger attendance toward stimuli higher in social significance like potential mates (Feinberg, 2008), which could be considered as an important aspect of relationship functioning not only in initiation but also in maintaining phases. The studies covered in this review seem to foster this interpretation: during later phases of relationship and especially in “rough” times, spouses seem to especially use f_0_ as a non-verbal signal in relationship-relevant information like expression of distress.

### FINDINGS WITH REGARD TO GENDER DIFFERENCES

Across age and interaction settings, gender differences were found in a number of studies. Biologically speaking, f_0_ is based on vocal cord length and tension. Given the physiological differences in body size and thus, throat length between men and women, gender differences in f_0_ emerge after puberty with females on average having significantly higher f_0_ scores than males (Titze, 1989). Two studies in this review (Baucom et al., 2012b; Weusthoff et al., 2013) explicitly investigated gender differences and as expected found higher f_0_ range scores for female than for male speakers. Except for Baucom et al. (2011) and Weusthoff et al. (2013), all studies investigating adult speakers included in this review found variables of interest to have significant associations with f_0_ range for female speakers only, or found different associations for men and women. Given that female partners are more likely to seek help with regard to problems and/or conflict in close personal relationships (O’Brien, 1988) it seems also plausible that gender differences could stem from different goals and resulting goal-oriented behavior in males and females (especially as no associations being significant for men only emerged). Finding the best partner to produce healthy offspring seems to be an important goal during selection of a potential mate and sex partner during courtship. Significant associations between perceived, and self-rated attractiveness and health, and f_0_ for both sexes during sexual selection (though due to endocrine reasons in different directions, with higher attractiveness and perceived health being associated to f_0_ scores in women and lower f_0_ scores in men; Puts, 2010) also hint at f_0_ being an important aspect in goal-relevant behavior.

### LIMITATIONS

The work reviewed in this manuscript has taken into account only three indicators of vocally encoded emotional arousal out of a wide variety of potential ones (Juslin and Scherer, 2005, pp. 103-104): f_0_ range, f_0_ mean, and f_0_-time-to-peak as a time-varying aspect of f_0_. This has happened for a number of reasons. As earlier work on vocally encoded emotional arousal has focused on f_0_ mean in a number of different research settings (i.e., f_0_ in speech of psychiatric patients, Tolkmitt et al., 1982; or emotion portrayal by professional actors, Banse and Scherer, 1996), it was chosen as the parameter of interest in the first study conducted on f_0_ in close personal relationships (Baucom et al., 2011) in order to enable comparisons to these published empirical findings. However, among the different f_0_ indices that can be calculated f_0_ range seems to bear a number of advantages in research on close personal relationships (see Juslin and Scherer, 2005 for a review), and was therefore chosen for the studies conducted later. f_0_ range is considered to be the cleanest vocal indicator of emotional arousal, and to depict the biggest amount of information on emotional arousal (Juslin and Scherer, 2005; Busso et al., 2009). Furthermore, it facilitates the interpretation of gender differences as it adjusts for potential individual differences by way of its calculation (subtracting the individual’s minimum f_0_ score from the individual’s f_0_ maximum score; Weusthoff et al., 2013). Nonetheless, it is possible that other indicators of vocally emotional arousal (e.g., jitter) might contain additional information on the nature of emotional arousal in close personal relationship communication.

Though biological reasons for differences in male and female voices are well-documented and have been discussed in this review, it remains unclear why expected sex differences for f_0_ indices and their associations with different variables of interest did not emerge consistently across studies. Particularly, none of the studies found significant gender differences in the associations between f_0_ mean (compared to studies using f_0_ range) and variables of interest. Future research should regard gender as a covariate in f_0_ research in close personal relationship but more detailed investigations on the details of gender differences in f_0_ indices are needed.

Investigating emotional arousal in human speech using f_0_ includes the drawback of participants being able to willingly influence and control f_0_ to a certain degree. Empirical evidence has shown that conscious manipulations can result in both elevated and lowered pitch levels that are identifiable by a communication partner (Kuenzel, 2000). However, given the arousing, stressful, and cognitively demanding situation of couple conflict, it is quite unlikely that participants in the studies reviewed made use of this hypothetical possibility.

### IMPLICATIONS FOR CLINICAL WORK AND FUTURE RESEARCH

As f_0_ seems to be able to display an individual’s internal levels of emotional arousal, other dyadic settings in which emotional arousal is considered to be important information could also benefit from research on vocally encoded arousal. During social support interactions between spouses, showing the interaction partner positive and helpful behaviors in order to help him or her to deal with stressful situations is the main goal. Social support is considered to consist of different positive and helpful behaviors being observable in communication between interaction partners leading to changes in individual cardiovascular, and endocrine functioning which influences individual health outcomes. Consistent with this model, lower levels of social support behavior have been empirically linked to higher levels of physiological indices of arousal (most often BP), and to poorer health outcomes in affected individuals (Uchino et al., 1996). Given the associations between BP, and HR with f_0_ in couple conflict, vocally encoded emotional arousal also seems to be important during social support interactions between spouses. However, in comparison to conflict discussions, more positive and less emotionally arousing behaviors seem be goal-relevant in social support and should therefore be displayed more often between interaction partners (Verhoftstadt et al., 2005). Links between f_0_ and variables of interest in social support behavior could therefore be of different direction and magnitude than the ones found in conflict discussions.

Communication Accomodation Theory (CAT; Giles and Coupland, 1991) describes changes in partners’ communication styles during a conversation leading to more, or less similitarity between the partners (convergence and divergence). Convergence can occur on different levels (e.g., pitch pattern, speech rate, or emotional expression), and has empirically been shown to occur in various dyads. Convergence seems to happen somewhat naturally but is associated with different outcomes depending on the interaction setting and other external factors in intimate relationships. Convergence seems to be a beneficial process during interactions asking for social support between partners. For example, high levels of emotional convergence (meaning similarity in emotional expressions between spouses) while sharing emotional events of one’s day are related to higher levels of concurrent and longitudinal relationship satisfaction, and higher relationship stability (Anderson et al., 2003). During couple conflict, however, convergence in emotional expression and behavior like the DW pattern have negative impacts on relationship functioning and outcomes (Lee et al., 2012; Baucom and Atkins, 2013). A closer look at potential reasons for these differential effects of convergence in couples’ communication could be a fruitful avenue for future research. Are the associations due to levels of relationship satisfaction (happy vs. unhappy couples), do they depend on the context of the interaction (social support vs. conflict), or do both factors and/or a third one contribute?

Vocal characteristics between talkshow host and guests have also been demonstrated to converge across the course of an interview. However, across different dyads, status and/or power seem to be an important factor influencing convergence: The conversation partner lower in status and power (having a greater “need for social approval”; Giles and Coupland, 1991, p. 73) exhibits more change in vocal and emotional expressions, moving in the direction of the more powerful partner (Gregory and Webster, 1996).

Power processes are also influential in couples communication (Baucom et al., 2011), and are interdependently linked to each other. For example, f_0_ in distressed spouses’ problem-solving discussions is known to converge in terms of magnitude: if one spouse is highly aroused, the other partner is also more likely to be highly aroused. Furthermore, this covariation seems to lead to problems in partner’s ability to regulate their own state of arousal to a comfortable level (Baucom and Atkins, 2013).

Given these findings, it seems likely that spouses’ f_0_ scores could also be associated with each other across speakers. Gottman and Notarius (2002, p.185) explicitly state that there is a “need for continued focus on sequences or patterns of interaction” in order to identify beneficial and harmful aspects of spousal communication. Sequential analyses of f_0_ could shed light on presence, magnitude, and direction of interdependent and cyclical aspects of emotional arousal in couple communication.

## Conflict of Interest Statement

The authors declare that the research was conducted in the absence of any commercial or financial relationships that could be construed as a potential conflict of interest.
